# Pediatric SARS-CoV-2 infection and development of anxiety and depression

**DOI:** 10.3389/fped.2025.1524617

**Published:** 2025-03-17

**Authors:** Frederick Dun-Dery, Jianling Xie, Roger Zemek, Kathleen Winston, Brett Burstein, Vikram Sabhaney, Jason Emsley, Jocelyn Gravel, April Kam, Ahmed Mater, Darcy Beer, Robert Porter, Gabrielle Freire, Naveen Poonai, Anne Moffatt, Simon Berthelot, Marina I. Salvadori, Deepti Reddy, Bruce Wright, Stephen B. Freedman

**Affiliations:** ^1^ Department of Pediatrics, Cumming School of Medicine, University of Calgary, Calgary, AB, Canada; ^2^ Section of Pediatric Emergency Medicine, Department of Pediatrics, Cumming School of Medicine, University of Calgary, Calgary, AB, Canada; ^3^ Department of Pediatrics, University of Ottawa, Children’s Hospital of Eastern Ontario, Ottawa, ON, Canada; ^4^ Department of Emergency Medicine, University of Ottawa, Children’s Hospital of Eastern Ontario, Ottawa, ON, Canada; ^5^ Division of Pediatric Emergency Medicine, Department of Pediatrics, Montreal Children’s Hospital, McGill University Health Centre, Montreal, QC, Canada; ^6^ Department of Epidemiology, Biostatistics, and Occupational Health, McGill University, Montreal, QC, Canada; ^7^ Department of Pediatrics, BC Children’s Hospital and BC Children’s Research Institute, University of British Columbia, Vancouver, BC, Canada; ^8^ Department of Pediatric Emergency Medicine, Centre Hospitalier Universitaire (CHU) Sainte-Justine, Université de Montréal, Montreal, QC, Canada; ^9^ Division of Emergency Medicine, Department of Pediatrics, McMaster Children’s Hospital, Hamilton, ON, Canada; ^10^ Department of Emergency Medicine, IWK Children’s Health Centre and Queen Elizabeth II Health Sciences Centre, Dalhousie University, Halifax, NS, Canada; ^11^ Section of Pediatric Emergency, Department of Pediatrics, Jim Pattison Children’s Hospital, University of Saskatchewan, Saskatoon, SK, Canada; ^12^ Department of Pediatrics and Child Health, The Children’s Hospital of Winnipeg, Children’s Hospital Research Institute of Manitoba, University of Manitoba, Winnipeg, MB, Canada; ^13^ Department of Pediatrics, Janeway Children’s Health and Rehabilitation Centre, NL Health Services, St John’s, NL, Canada; ^14^ Division of Emergency Medicine, Department of Pediatrics, Hospital for Sick Children, Faculty of Medicine, University of Toronto, Toronto, ON, Canada; ^15^ Department of Pediatrics, Schulich School of Medicine & Dentistry, London, ON, Canada; ^16^ Department of Internal Medicine, Schulich School of Medicine & Dentistry, London, ON, Canada; ^17^ Department of Epidemiology & Biostatistics, Schulich School of Medicine & Dentistry, London, ON, Canada; ^18^ Department of Pediatrics, Kingston Health Sciences Centre, Queen’s University, Kingston, ON, Canada; ^19^ Département de Médecine de Famille et de Médecine d’Urgence, CHU de Québec-Université Laval, Québec City, QC, Canada; ^20^ Department of Pediatrics, Faculty of Medicine, McGill University, Montreal, QC, Canada; ^21^ Clinical Research Unit, Children’s Hospital of Eastern Ontario Research Institute, University of Ottawa, Ottawa, ON, Canada; ^22^ Department of Pediatrics, Women’s and Children’s Research Institute, University of Alberta, Edmonton, AB, Canada; ^23^ Sections of Pediatric Emergency Medicine and Gastroenterology, Departments of Pediatrics and Emergency Medicine, Cumming School of Medicine, University of Calgary, Calgary, AB, Canada

**Keywords:** SARS-CoV-2, COVID, anxiety, depression, children, emergency department (ED)

## Abstract

**Objective:**

It remains unclear whether emerging mental health concerns in children infected with SARS-CoV-2 are a direct result of the infection or due to the indirect effects of the pandemic. Therefore, we sought to assess the frequency of new diagnoses of anxiety and/or depression among children diagnosed with and without SARS-CoV-2 infection who were tested in pediatric emergency departments.

**Methods:**

A prospective cohort study with 6- and 12-month follow-ups was conducted across 14 Canadian tertiary-care pediatric emergency departments of the Pediatric Emergency Research Canada (PERC) network. The study included children aged <18 years who were tested for SARS-CoV-2 infection between August 2020 and February 2022. The primary outcome was the diagnosis of anxiety and/or depression reported during follow-up. The surveys incorporated a modified version of the International Severe Acute Respiratory and Emerging Infection Consortium (ISARIC) Long-COVID Pediatric Questionnaire.

**Results:**

Among the participants who were eligible for 6- and 12-month follow-ups, 64.7% (268/414) of SARS-CoV-2-positive and 71.9% (743/1,033) of SARS-CoV-2-negative participants completed follow-up at these time points, respectively. The median age was 7.0 [inter-quartile range (IQR): 5.0–11.0] years, and 54.2% (548/1,011) were male. New diagnoses of anxiety and/or depression reported on either survey did not differ significantly between test-positive (4.1%, 11/268) and test-negative (2.8%; 21/743) participants [difference = 1.3% (95% CI: −1.3 to 4.2)]. There was a higher prevalence of new diagnoses of anxiety and/or depression among SARS-CoV-2-negative participants aged ≥12 years relative to those aged <12 years [8.7% (13/149) vs. 1.3% (8/594); difference = 7.4%; 95% CI of the difference = 3.0–12.5], but not among SARS-CoV-2-positive participants [4.4% (2/45) vs. 4.0% (9/223); difference = 0.4%; 95% CI of the difference = −5.6 to 9.4]. At 6 or 12 months, SARS-CoV-2-positive participants were more likely to experience confusion and/or lack of concentration, abdominal pain, and insomnia.

**Conclusions:**

Although no association was found between SARS-CoV-2 infection and new diagnoses of anxiety and/or depression, SARS-CoV-2-positive participants were more likely to experience confusion/lack of concentration, abdominal pain, and insomnia. This finding, in the context of an increased prevalence of new diagnoses of anxiety and depression, underscores the impacts of societal changes on the mental health of children. Our finding that some non-specific symptoms were more frequently reported by SARS-CoV-2-positive participants emphasizes the need for further investigation of the underlying pathophysiologic mechanisms.

## Introduction

The COVID-19 pandemic has posed significant challenges to mental health, particularly among children and adolescents (1). A substantial body of literature has emerged, suggesting that adolescents have experienced a significant increase in psychological distress manifesting as depression and anxiety (2). According to the 2021 Adolescent Behaviors and Experiences Survey, 37% of US high school students reported poor mental health during the pandemic, with 20% considering and 9.0% attempting suicide in the preceding year (3).

The long-term effects of COVID-19, including potential mental health issues, are a growing public health concern (4). Although the prevalence of mental health conditions increased in children and adolescents during the COVID-19 pandemic (4–7), it remains unclear whether this is due to the direct effects of SARS-CoV-2 infection itself, the broader social and situational context of the pandemic, or a combination of both (8). Reviews have called for further research to determine whether the neuropsychiatric symptoms reported in children with the post-COVID-19 condition are a result of COVID-19 infection; stress, anxiety, and behavioral changes related to public health restrictions imposed to mitigate the spread of the SARS-CoV-2 virus; or other societal influences (9).

Both adult (10–12) and pediatric (13) electronic health record cohort studies have demonstrated an increased incidence of neurologic and psychiatric diagnoses following SARS-CoV-2 infection (11, 12). Although few prospective studies that include test-negative controls have addressed this question, a national, cross-sectional study conducted in Denmark found that although SARS-CoV-2-positive participants were more likely to have chronic symptoms, SARS-CoV-2-negative controls had lower quality of life scores, including the emotional, social and school functioning subscales (14).

SARS-CoV-2 may affect brain function by binding to angiotensin-converting enzyme type 2 receptors present in the central nervous system (15). In addition to the potential impact of direct viral infection on neural cells, SARS-CoV-2 infection may also lead to depression through an indirect neuroinflammatory immune response, i.e., cytokine storm (16), which involves the production of interleukin 1β and interleukin 6 (17). Cytokines can lead to depression in various ways including hyperactivation of the hypothalamic–pituitary–adrenal axis which can result in neurotoxicity and neurodegeneration which are associated with the development of depression (18). In addition, other etiologies of depression in SARS-CoV-2-infected children include chronic physical symptoms (19), invalidation by healthcare providers (20), isolation from peers (21), and school absenteeism (22).

Although COVID-19 pandemic restrictions have generally been removed, the SARS-CoV-2 virus continues to circulate (23). Given the concerns regarding the direct neuropsychiatric effects of SARS-CoV-2 infection, it remains important to distinguish between the direct impact of SARS-CoV-2 infection and the indirect effects of the COVID-19 pandemic on mental health (24). Such evaluations must differentiate the direct effects of infection from those due to the situational context created by the pandemic. To address these knowledge gaps, we sought to determine if children who tested positive for SARS-CoV-2 were more likely to be diagnosed with anxiety and/or depression over the subsequent 12 months compared to those who tested negative.

## Materials and methods

### Study design and setting

This study is a secondary analysis of a prospective cohort study of participants recruited from 14 Pediatric Emergency Research Canada (PERC) tertiary-care pediatric emergency departments (EDs) (25) between August 2020 and February 2022. Research ethics board approval was obtained at all participating institutions (Supplementary Table S1), and informed oral consent was obtained along with participant assent as per institutional policy. Study findings are reported as per the Strengthening the Reporting of Observational Studies in Epidemiology guidelines (26).

### Participants and recruitment

We recruited children aged <18 years who underwent SARS-CoV-2 testing in a participating ED. Specimen collection was performed at the treating physician's discretion and/or per institutional policy. Collected specimens were analyzed using nucleic acid amplification approaches as determined by the local laboratory. We excluded participants <4 years of age as mental health disorders are less often diagnosed in younger children (27). Participants who neither spoke nor understood French and/or English were also excluded. The follow-up surveys were added to the study protocol on 1 November 2021; participants enrolled >12 months before this date were deemed ineligible. We excluded participants who reported seeking support from a mental health specialist (e.g., psychiatrist, psychologist, social worker, or counselor) prior to the onset of the COVID-19 pandemic.

To enable the identification of potentially eligible participants, team members received a list of all children tested daily. Research assistants phoned the caregivers of SARS-CoV-2-positive children first, then those who tested negative, starting with the test performed earliest on each day. This approach was adopted to minimize selection bias should the number of potentially eligible children exceed the capacity of the research team. During the consenting process, we limited the information provided about the rationale for conducting long-term follow-up to the following sentence: “Because of what we have learned about the long-term consequences of COVID infection in children, we have been asked by the Public Health Agency of Canada to collect additional outcome data 6 and 12 months following the emergency department visit.”

### Outcomes measures

The primary outcome was a reported diagnosis of anxiety and/or depression in the 6- and/or 12-month follow-up surveys. These timepoints were selected as it was *a priori* determined that the comprehensive follow-up surveys would be administered at those time points. Secondary outcomes focused on non-specific symptoms, which we sought to compare between children infected and uninfected with SARS-CoV-2. This comparison enabled us to determine if the development of symptoms was more common among infected children compared to uninfected children, thus permitting an assessment of symptoms as a manifestation of the direct effects of SARS-CoV-2 infection vs. being more a reflection of the effects of the lockdowns, school closures, and limitations of leisure activities (28). We sought to compare the reporting of any of the following symptoms (as composite and individual measures) at the 6- and 12-month surveys: headache, dizziness, syncope, palpitations, chest pain, abdominal pain, nausea, balance problems, myalgias, visual concerns, tremors, paresthesia, concentration challenges, insomnia, hypersomnia, fatigue, and poor appetite.

### Data collection

Data were collected as soon as possible following the index ED visit and via caregiver completion of surveys at 6- and 12-month follow-ups. A medical record review was performed to classify SARS-CoV-2 status based on the result of the test performed at the index ED visit and any additional tests performed during the subsequent 14 days. The 6- and 12-month follow-up surveys included a modified version of the International Severe Acute Respiratory and Emerging Infection Consortium (ISARIC) Long-COVID Pediatric Questionnaire (Supplementary Table S2) (29). This questionnaire has become the gold standard tool used by leading investigations into the assessment of long-COVID symptoms in the pediatric population (30).

### Definitions

To meet the study's primary outcome definition, caregivers had to respond yes to specific questions about their child receiving a new diagnosis of anxiety or depression since their index ED visit. We defined exposure status (i.e., SARS-CoV-2-positive/negative) based on detecting SARS-CoV-2 on a swab specimen collected from the nares, nasopharynx, or oral cavity at the index ED visit or within 14 days thereafter. Children who tested negative constituted the control group. Hospitalization status was defined by admission to a hospital at the index ED visit or within the subsequent 14 days (31). The approach to testing and reporting of variants differed across participating institutions. When variant testing was performed, that result was used. However, when variant testing was either not performed or was inconclusive, the SARS-CoV-2 variant was imputed following established methods (32).

### Sample size

Pooled estimates obtained in the first year of the COVID-19 pandemic suggest that depression and anxiety symptoms doubled compared to pre-pandemic levels (33). Our estimate of the baseline rate for new diagnoses of anxiety or depression comes from a scoping review of US children, which showed that 10% of children and adolescents received mental health services in the past year, as reported by their parents (34). Based on earlier analyses of our cohort, we anticipated having four SARS-CoV-2-negative controls per case (32). Thus, to have 90% power with an alpha of 0.05, we required 684 SARS-CoV-2-negative and 171 SARS-CoV-2-positive participants to identify a doubling in the prevalence of mental health conditions from 10% to 20%.

### Statistical analysis

We summarized participant demographic and clinical characteristics using descriptive statistics. For the primary outcome of new mental health diagnoses, and for the secondary outcomes of reporting individual symptoms, we used Chi-square tests and Fisher's exact tests for comparisons between SARS-CoV-2-positive and SARS-CoV-2-negative groups. The Wald test was used to obtain the 95% CI of the difference between proportions, and the Agresti-Caffo approach was used when the event was rare (*n* < 20) (35). *P*-values obtained from unadjusted bivariate analyses were adjusted via the Benjamini–Hochberg approach for multiple comparisons (36). We also conducted stratified analyses by participant age and diagnosis (i.e., anxiety and depression).

We did not perform multiple imputations to address missing data for the predictor and outcome variables as the missing at-random assumption is unlikely to be true. All analyses were two-sided, and statistical significance was defined by *P* < 0.05. Analyses were performed using SPSS Statistics for Windows, version 29 (IBM Corporation), and R-4.3.2. Data were analyzed from 26 February 2024 to 31 March 2024.

## Results

### Participant characteristics

**Figure 1:**
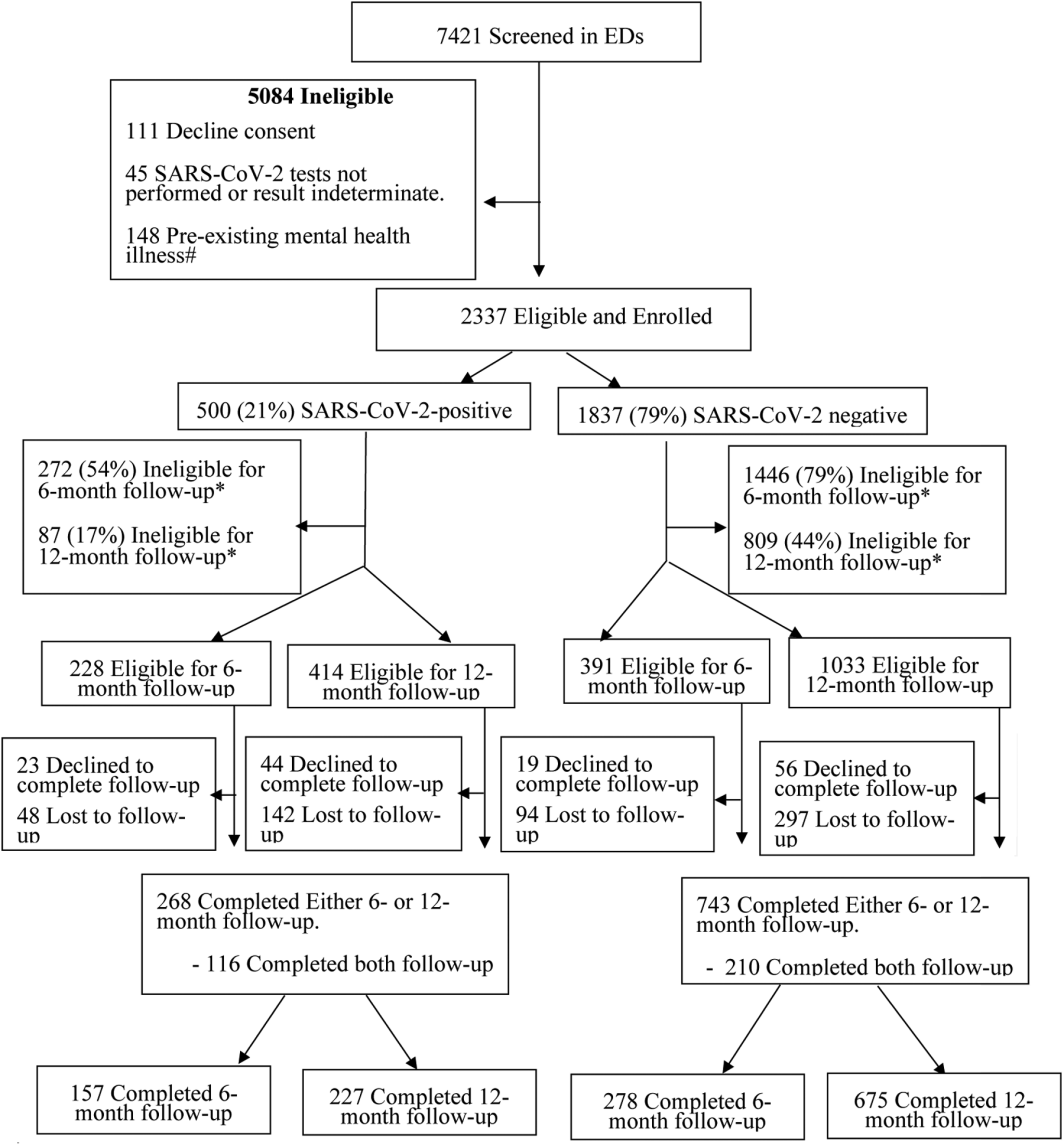
Flow diagram of participants from index emergency department enrolment visit to 12-month follow-up. #Participants who reported seeking support from a mental health specialist (e.g., psychiatrist, psychologist, social worker, and counselor) prior to the onset of the COVID-19 pandemic were excluded. *Had passed the 6- and/or 12-month follow-up survey time windows.

A total of 7,421 children were screened, and 2,337 (31.5%) met the inclusion criteria for the analysis, of whom 21.4% (500/2,337) and 78.6% (1,837/2,337) were SARS-CoV-2-positive and SARS-CoV-2-negative, respectively. Among SARS-CoV-2-positive participants eligible for 6- and 12-month follow-ups, 68.9% (157/228) and 54.8% (227/414) completed follow-up at these time points, respectively. Among SARS-CoV-2-negative participants, completion rates were 56.6% (278/491) and 65.3% (675/1,033), respectively (Figure 1). Participants who completed vs. those who did not complete either the 6- or 12-month survey differed with respect to age, COVID-19 vaccination status, number of symptoms at baseline, and SARS-CoV-2 variant (Supplementary Table S3). Follow-up was completed at either of these time points by 64.7% (268/414) and 71.9% (743/1,033), of SARS-CoV-2-positive and negative participants, respectively.

Participant median age was 7 [inter-quartile range (IQR): 5–11] years, 54.2% (548/1,011) were male, 44.8% (448/999) self-identified as White, 12.2% (123/1,011) were hospitalized, and 1% (10/1,006) had a severe outcome (Table 1). Fifty-three percent (396/743) of SARS-CoV-2-negative participants were enrolled during the transmission periods of the wild-type and alphavirus subtypes, while 25.4% of SARS-CoV-2-positive participants were enrolled during the same period.

**Table 1:** Baseline characteristics, stratified by SARS-CoV-2 test outcome, inclusive of all participants that completed either the 6- or 12-month follow-up surveys.

Baseline characteristic	All *N* = 1,011	SARS-CoV-2-negative *N* = 743	SARS-CoV-2-positive *N* = 268
Age, years, median (IQR)	7 (5–11)	7 (5–11)	8 (6–10)
Age group, *n* (%)			
4–12	817/1,011 (80.8)	594/743 (79.9)	223/268 (83.2)
12–18	194/1,011 (19.2)	149/743 (20.1)	45/268 (16.8)
Sex, male, *n* (%)	548/1,011 (54.2)	396/743 (53.3)	152/268 (56.7)
Race/ethnicity, *n* (%)			
White	448/999 (44.8)	358/735 (48.7)	90/264 (34.1)
Mixed	132/999 (13.2)	104/735 (14.1)	28/264 (10.6)
Middle Eastern	109/999 (10.9)	65/735 (8.8)	44/264 (16.7)
South Asian	80/999 (8.0)	52/735 (7.1)	28/264 (10.6)
Black	79/999 (7.9)	46/735 (6.3)	33/264 (12.5)
East Asian	43/999 (4.3)	32/735 (4.4)	11/264 (4.2)
Latin American	46/999 (4.6)	32/735 (4.4)	14/264 (5.3)
Southeast Asian	38/999 (3.8)	26/735 (3.5)	12/264 (4.5)
Indigenous	24/999 (2.4)	20/735 (2.7)	4/264 (1.5)
Asthma, *n* (%)	188/1,009 (18.6)	145/743 (19.5)	43/266 (16.2)
Pre-existing chronic condition^a^, *n* (%)	190/1,009 (18.8)	135/743 (18.2)	55/266 (20.7)
Indigenous, *n* (%)	64/1,004 (6.4)	54/739 (7.3)	10/265 (3.8)
Number of symptoms at baseline, median (IQR)	6 (3, 8)	5 (3, 8)	6 (4, 9)
COVID vaccination received prior to index visit, *n* (%)^b^	120/662 (18.1)	69/444 (15.5)	51/218 (23.4)
Hospitalized for the acute illness, *n* (%)^d^	123/1,011 (12.2)	90/743 (12.1)	33/268 (12.3)
Severe acute illness outcome, *n* (%)^d^^,^^e^	10/1,006 (1.0)	6/742 (0.8)	4/264 (1.5)
Variant, *n* (%)^c^			
Wild type	266/1,011 (26.3)	236/743 (31.8)	30/268 (11.2)
Alpha	198/1,011 (19.6)	160/743 (21.5)	38/268 (14.2)
Delta	384/1,011 (38.0)	288/743 (38.8)	96/268 (35.8)
Omicron	163/1,011 (16.1)	59/743 (7.9)	104/268 (38.8)

^a^ Excluding asthma.

^b^ The question was implemented in the study on 11 June 2021, approximately 10 months after the study was officially launched.

^c^ For SARS-CoV-2-negative group coded as: wild type (before 18 April 2021), alpha (18 April 2021–26 June 2021), delta (27 June 2021–11 December 2021), and omicron (12 December 2021–present day).

^d^ At or within 14 days of the index ED visit.

^e^ A severe outcome reflected the occurrence of a life-threatening complication or performance of an intervention required to address a potentially life-threatening event (31).

### Primary outcome

A new diagnosis of anxiety and/or depression provided by a healthcare professional reported by caregivers at the 6- and/or 12-month surveys did not differ between SARS-CoV-2-positive (4.1%, 11/268) and SARS-CoV-2-negative participants (2.8%; 21/743) [difference = 1.3% (95% CI: −1.3 to 4.2); Table 2]. Among participants aged 4–12 years, the percentage difference between SARS-CoV-2-positive and SARS-CoV-2-negative participants was 2.7% (95% CI: 0.1–5.8; adjusted *P* = 0.22). Among those aged 12–18 years of age, there was no association between SARS-CoV-2 test status and a new diagnosis of anxiety or depression (−4.3% SARS-CoV-2-positive relative to negative; 95% CI: −11.3 to 5.5). Although the prevalence of new diagnoses of anxiety or depression was greater among SARS-CoV-2-negative participants aged ≥12 years relative to those aged <12 years [8.7% (13/149) vs. 1.3% (8/594); difference = 7.4%; 95% CI of the difference = 3.0–12.5], there was no such difference among SARS-CoV-2-positive participants [4.4% (2/45) vs. 4.0% (9/223); difference = 0.4%; 95% CI of the difference = −5.6 to 9.4].

**Table 2:** Primary outcomes based on index SARS-CoV-2 emergency department index visit test result status.

Outcome	SARS-CoV-2-negative	SARS-CoV-2-positive	% Difference (95% CI)	Unadjusted odds ratio (95% CI)	Adjusted *P*-value^a^
Anxiety and/or depression	21/743 (2.8)	11/268 (4.1)	1.3 (−1.3 to 4.2)	1.5 (0.7–3.1)	0.56
Depression	6/743 (0.8)	4/268 (1.5)	0.7 (−0.8 to 2.7)	1.9 (0.5–6.7)	0.68
Anxiety	21/743 (2.8)	9/268 (3.4)	0.5 (−1.8 to 3.3)	1.2 (0.5–2.6)	0.68
Age 4–12 years					
Anxiety and/or depression	8/594 (1.3)	9/223 (4.0)	2.7 (0.1–5.8)	3.1 (1.2–8.1)	0.22
Depression	1/594 (0.2)	2/223 (0.9)	0.7 (−0.6 to 2.6)	5.4 (0.5–59.5)	0.44
Anxiety	8/594 (1.3)	8/223 (3.6)	2.2 (−0.3 to 5.2)	2.7 (1.0–7.4)	0.22
Age 12–18 years					
Anxiety and/or depression	13/149 (8.7)	2/45 (4.4)	−4.3 (−11.3 to 5.5)	0.5 (0.1–2.2)	0.68
Depression	5/149 (3.4)	2/45 (4.4)	1.1 (−5.2 to 10.1)	1.3 (0.3–7.2)	0.68
Anxiety	13/149 (8.7)	1/45 (2.2)	−6.5 (−12.4 to 2.4)	0.2 (0.03–1.9)	0.44

^a^ *P*-values were adjusted via the Benjamini–Hochberg approach for multiple comparisons.

### Secondary outcomes

**Figure 2:**
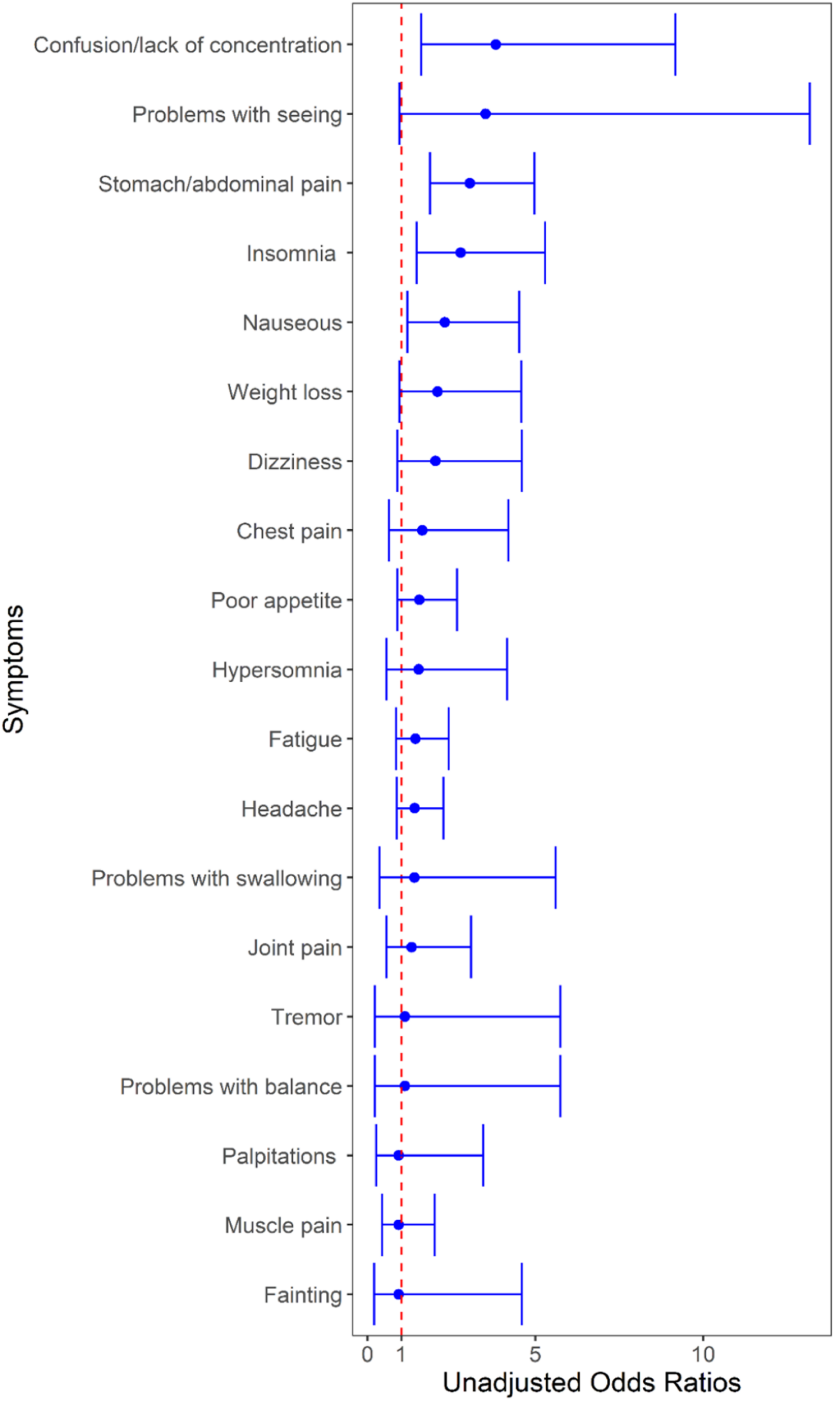
Unadjusted odds of having individual symptoms at 6- and/or 12-month follow-up based on index emergency department visit SARS-CoV-2 test result. The region to the right of the red dotted line indicates an increased risk associated with a positive test. Each line in the forest plot represents a different symptom. The blue dot marks the unadjusted odds ratio (OR) for each symptom in children who tested positive for SARS-CoV-2 compared to those who tested negative. The horizontal blue lines through the dots represent the 95% confidence intervals for the OR. The red vertical dashed line represents the line of no effect—a reference point for determining the significance of the results.

At 6 and/or 12 months, 28.7 (77/268) and 21.3% (158/743) of SARS-CoV-2-positive and SARS-CoV-2-negative participants, respectively, reported any ongoing symptoms (difference = 7.5%; 95% CI: 1.3, 13.6, *p* = 0.09; Figure 2, Table 3A). SARS-CoV-2-positive participants were more likely to report experiencing confusion/lack of concentration, abdominal pain, and insomnia. When analyzed by individual time point (i.e., 6 and 12 months separately), the only symptom that differed between groups was abdominal pain at 12 months which was more common among SARS-CoV-2-positive participants (OR = 2.7, 95% CI: 1.6–4.8, adjusted *P* = 0.007; Supplementary Tables S4, S5). When participants were grouped based on the diagnosis of anxiety and/or depression during the follow-up period, nearly all symptoms were more common among those with anxiety and/or depression (Table 3B). The symptoms with the greatest between-group absolute percent differences were abdominal pain (31.6%), headache (26.8%), poor appetite (22.9%), muscle pain (22.1%), and joint pain (20.0%).

**Table 3:** Secondary outcomes reported at 6- and/or 12-months stratified by the index ED visit SARS-CoV-2 test.

Symptomatology	All *N* = 1,011	SARS-CoV-2-negative *N* = 743	SARS-CoV-2-positive *N* = 268	Difference of % (95% CI)	Unadjusted odds ratio (95% CI)	Adjusted *p*-value^a^
Anxiety and/or depression	32/1,011 (3.2)	21/743 (2.8)	11/268 (4.1)	1.3 (−1.3 to 4.2)	1.47 (0.7–3.1)	0.53
Anxiety	30/1,011 (3.0)	21/743 (2.8)	9/268 (3.4)	0.5 (−1.8 to 3.3)	1.2 (0.5–2.6)	0.89
Depression	10/1,011 (1)	6/743 (0.8)	4/268 (1.5)	0.7 (−0.84 to 2.7)	1.7 (0.52–6.7)	0.53
Any symptoms	235/1,011 (23.2)	158/743 (21.3)	77/268 (28.7)	7.5 (1.3–13.6)	1.5 (1.1–2.1)	0.09
Abdominal pain	70/1,011 (6.9)	35/743 (4.7)	35/268 (13.1)	8.4 (4.0–12.7)	3.0 (1.9–5)	<0.001
Chest pain	19/1,011 (1.9)	12/743 (1.6)	7/268 (2.6)	1.0 (−1.0 to 3.5)	1.6 (0.6–4.2)	0.69
Confusion/lack of concentration	21/1,011 (2.1)	9/743 (1.2)	12/268 (4.5)	3.3 (0.8–6.2)	3.8 (1.6–9.2)	0.02
Dizziness	24/1,011 (2.4)	14/743 (1.9)	10/268 (3.7)	1.9 (−0.5 to 1.9)	2.0 (0.9–4.6)	0.27
Fainting	8/1,011 (0.8)	6/743 (0.8)	2/268 (0.7)	−0.1 (−1.26 to 1.6)	0.9 (0.2–4.6)	>0.99
Fatigue	66/1,011 (6.5)	44/743 (5.9)	22/268 (8.2)	2.3 (−1.3 to 6.2)	1.4 (0.88–2.4)	0.40
Headache	85/1,011 (8.4)	57/743 (7.7)	28/268 (10.4)	2.8 (−1.21 to 7.1)	1.40 (0.9–2.3)	0.40
Hypersomnia	17/1,011 (1.7)	11/743 (1.5)	6/268 (2.2)	0.8 (−1.1 to 3.1)	1.5 (0.6–4.2)	0.87
Insomnia	39/1,010 (3.9)	20/743 (2.7)	19/267 (7.1)	4.4 (1.3–8.0)	2.8 (1.45–5.3)	0.02
Joint pain	25/1,011 (2.5)	17/743 (2.3)	8/268 (3.0)	0.7 (−1.5 to 3.3)	1.3 (0.6–3.1)	0.89
Muscle pain	36/1,011 (3.6)	27/743 (3.6)	9/268 (3.4)	−0.3 (−2.7 to 2.6)	0.9 (0.4–2.0)	>0.99
Nausea	36/1,010 (3.6)	20/743 (2.7)	16/267 (6.0)	3.3 (0.4–6.6)	2.3 (1.2–4.5)	0.09
Palpitations	12/1,011 (1.2)	9/743 (1.2)	3/268 (1.1)	−0.1 (−1.5 to 1.8)	0.9 (0.3–3.4)	0.99
Poor appetite	60/1,011 (5.9)	39/743 (5.2)	21/268 (7.8)	2.6 (−0.9 to 6.4)	1.5 (0.9–2.7)	0.32
Problems seeing	9/1,011 (0.9)	4/743 (0.5)	5/268 (1.9)	1.3 (−0.3 to 3.4)	3.5 (0.9–13.2)	0.22
Problems with balance	7/1,011 (0.7)	5/743 (0.7)	2/268 (0.7)	0.1 (−1.1 to 1.7)	1.1 (0.2–5.8)	>0.99
Problems with swallowing	9/1,011 (0.9)	6/743 (0.8)	3/268 (1.1)	0.3 (−1.1 to 2.1)	1.4 (0.3–5.6)	0.89
Tingling	2/1,011 (0.2)	0/743 (0)	2/268 (0.7)	0.8 (−0.3 to 2.3)	N/A	0.22
Tremor	7/1,011 (0.7)	5/743 (0.7)	2/268 (0.7)	0.1 (−1.10 to 1.7)	1.11 (0.2–5.8)	>0.99
Weight loss	26/1,011 (2.6)	15/743 (2.0)	11/268 (4.1)	2.1 (−0.4 to 5.0)	2.1 (0.9–4.6)	0.22
Secondary outcomes were reported at 6 and/or 12 months stratified by participants who developed anxiety and/or depression and those who did not develop						
Symptomatology	All *N* = 1,011	Did not develop anxiety/depression *N* = 979	Developed anxiety/depression *N* = 32	Difference of % (95% CI)	Unadjusted odds ratio (95% CI)	Adjusted *p*-value^a^
Any symptoms	235/1,011 (23.2)	211/979 (21.6)	24/32 (75.0)	53.4 (36.9–67.0)	10.9 (4.8–24.7)	<0.001
Abdominal pain	70/1,011 (6.9)	58/979 (5.9)	12/32 (37.5)	31.6 (15.8–48.6)	9.5 (4.4–20.4)	<0.001
Chest pain	19/1,011 (1.9)	16/979 (1.6)	3/32 (9.4)	7.7 (−0.8 to 20.9)	6.2 (1.7–22.6)	0.03
Confusion/lack of concentration	21/1,011 (2.1)	16/979 (1.6)	5/32 (15.6)	14.0 (3.1–28.8)	11.1 (3.8–32.6)	0.001
Dizziness	24/1,011 (2.4)	19/979 (1.9)	5/32 (15.6)	13.7 (2.8–28.5)	9.4 (3.3–26.9)	0.001
Fainting	8/1,011 (0.8)	6/979 (0.6)	2/32 (6.3)	5.6 (−1.4 to 17.7)	10.8 (2.1–55.8)	0.03
Fatigue	66/1,011 (6.5)	59/979 (6.0)	7/32 (21.9)	15.8 (3.1–31.7)	4.4 (1.8–10.5)	0.005
Headache	85/1,011 (8.4)	74/979 (7.6)	11/32 (34.4)	26.8 (11.5–43.8)	6.4 (3.0–13.8)	<0.001
Hypersomnia	17/1,011 (1.7)	11/979 (1.1)	6/32 (18.8)	17.6 (5.8–33.0)	20.3 (7.0–59.1)	<0.001
Insomnia	39/1,010 (3.9)	32/978 (3.3)	7/32 (21.9)	18.6 (5.9–34.5)	8.3 (3.3–20.5)	<0.001
Joint pain	25/1,011 (2.5)	18/979 (1.8)	7/32 (21.9)	20.0 (7.3–35.9)	14.9 (5.7–39.0)	<0.001
Muscle pain	36/1,011 (3.6)	28/979 (2.9)	8/32 (25.0)	22.1 (8.6–38.4)	11.3 (4.7–27.4)	<0.001
Nausea	36/1,010 (3.6)	29/978 (3.0)	7/32 (21.9)	18.9 (6.2–34.8)	9.2 (3.7–22.9)	<0.001
Palpitations	12/1,011 (1.2)	7/979 (0.7)	5/32 (15.6)	14.9 (4.0–29.7)	25.7 (7.7–86.2)	<0.001
Poor appetite	60/1,011 (5.9)	51/979 (5.2)	9/32 (28.1)	22.9 (8.7–39.4)	7.1 (3.1–16.2)	<0.001
Problems seeing	9/1,011 (0.9)	7/979 (0.7)	2/32 (6.3)	5.5 (−1.5 to 17.6)	9.3 (1.8–46.4)	0.04
Problems with balance	7/1,011 (0.7)	6/979 (0.6)	1/32 (3.1)	2.5 (−2.8 to 13.1)	5.2 (0.6–44.8)	0.21
Problems with swallowing	9/1,011 (0.9)	8/979 (0.8)	1/32 (3.1)	2.3 (−3.0 to 12.9)	3.9 (0.5–32.3)	0.25
Tingling	2/1,011 (0.2)	1/979 (0.1)	1/32 (3.1)	3.0 (−2.2, to 13.6)	31.5 (1.9–516.1)	0.07
Tremor	7/1,011 (0.7)	5/979 (0.5)	2/32 (6.3)	5.7 (−1.3 to 17.8)	13.0 (2.4–69.7)	0.03
Weight loss	26/1,011 (2.6)	24/979 (2.5)	2/32 (6.3)	3.8 (−3.3 to 15.9)	2.7 (0.6–11.7)	0.21

^a^ *P*-value**s** were obtained from Chi-square or Fisher's exact tests as appropriate; *P*-values were adjusted via the Benjamini–Hochberg approach for multiple comparisons.

## Discussion

In this pan-Canadian prospective cohort study of children aged 4–18 years, there was no association between SARS-CoV-2 infection and a new diagnosis of anxiety and/or depression at 6- and/or 12-month follow-ups. Although subgroup analyses based on age identified no differences between groups based on SARS-CoV-2 test status, among SARS-CoV-2-negative children, a greater proportion of teenagers (i.e., ≥12 years) relative to younger participants (4–12 years) received a new diagnosis of anxiety and/or depression. During follow-up, SARS-CoV-2-positive participants were more likely to report experiencing confusion/lack of concentration, abdominal pain, and insomnia.

The prevalence of mental health disorders in children and adolescents had been increasing during the pre-COVID-19 pandemic period with several United States-based pediatric health organizations declaring a national state of emergency in children's mental health (37). Between 2009 and 2019, feelings of sadness or hopelessness increased among high school students by 40% (38) and the suicide rate among individuals aged 10–24 years increased by 57% (39). Moreover, although 13%–20% of children living in the United States experience mental illness annually (40), 50% do not receive adequate treatment. In addition, anxiety and depression are some of the most common disorders affecting 10% and 20% of children aged 3–17 years and 12–17 years, respectively (34).

Posttraumatic stress disorder (PTSD) is a complex and severe mental disorder that is estimated to occur in 10% of the US population at least once during their lifetime (41). Emerging evidence indicates that children and adolescents are more susceptible to the adverse impacts of traumatic events such as infectious disease pandemics (42–44). The COVID-19 pandemic had the potential to trigger PTSD in children due to the imposition of quarantines, school closures, and lockdowns which led to an increase in screen time in children and youth (45, 46). The development of PTSD has been further exacerbated by the emergence of concerning diseases in children and youth such as the post-COVID-19 condition and the multisystem inflammatory syndrome (47). Common symptoms of PTSD in adolescents, which are important to monitor for following traumatic events, include anxiety and depression (48).

Understanding the true prevalence of the post-COVID-19 condition in children remains a challenge, particularly because subjective neuropsychiatric symptoms such as depression, anxiety, and cognitive deficits are prominent complaints among children that can arise from many etiologies (4). Although some reports state that 10%–20% (4) of children with COVID-19 experience chronic sequelae, recent studies with robust methodologic designs, including control groups, report much lower absolute increased risks (49, 50). An electronic health record retrospective cohort study (i.e., ICD-10-CM based) that included 659,286 children, reported a 3.7% increased prevalence of post-acute sequelae of infection among SARS-CoV-2-positive children compared with SARS-CoV-2-negative controls (51). Of particular note, the authors reported an association between prior SARS-CoV-2 infection and receipt of mental health treatment [adjusted hazard ratio (aHR): 1.6; 95% CI: 1.5–1.8] and anxiety symptoms (aHR: 1.3; 95% CI: 1.1–1.6) (51). In a nationwide cohort study conducted in Denmark including over 115,000 children, SARS-CoV-2-positive children were 0.8% more likely to report symptoms lasting >4 weeks (52). Data collection, which employed a questionnaire, revealed that children in the control group were more likely to report numerous symptoms including concentration difficulties, headaches, muscle and joint pains, cough, nausea, diarrhea, and fever. Although in previous analyses of this cohort (53, 54), we reported a low but detectable increased frequency of the post-COVID-19 condition, in this study, we report no statistical association between SARS-CoV-2 infection and new diagnoses of anxiety and/or depression. This may be due to our smaller sample size which may not have permitted the detection of small effect sizes. As such, we did find a small but not statistically significant increase in new diagnoses of anxiety and/or depression (1.3% (95% CI: −1.3 to 4.2) and similar results in relation to many of the individual secondary outcome symptoms.

In a nationally representative cross-sectional Canadian survey, 37%, 34%, and 29% of children and youth self-reported increased symptoms of anxiety, irritability, and mood disorders during the pandemic, respectively (55). The increase in the prevalence of these symptoms highlights the need to differentiate the direct effects of SARS-CoV-2 infection from those of the societal impacts of the pandemic itself. In our cohort, we previously reported the prevalence of the post-COVID-19 condition at 12 months was 0.5% greater among SARS-CoV-2-positive compared to SARS-CoV-2-negative children (53). Our current study, which focuses directly on mental health diagnoses, identified no difference between groups with respect to this subset of conditions.

We did find that confusion, concentration difficulties, insomnia, and abdominal pain were more common among test-positive children. Although recurrent abdominal pain has a prevalence of 14% in the pediatric population (56), and frequently is present in the absence of concomitant anxiety and depression, concentration difficulties (57) and insomnia (58) are commonly associated with the presence of anxiety and depression. The mechanism by which SARS-CoV-2 infection causes chronic abdominal pain remains poorly understood (59), but it may be associated with the neurocognitive symptoms seen in individuals with the post-COVID-19 condition. It is hypothesized that SARS-CoV-2 damages both cerebral blood vessels (60) and the intestinal wall (61) by binding to angiotensin-converting enzyme 2 receptors. This leads to cytokine production (62) which can compromise the brain's neurovascular unit and the intestinal barrier, thereby increasing permeability to harmful substances. The latter are hypothesized to be produced by intestinal microbiota which flourish during a period of post-SARS-CoV-2 infection dysbiosis (63). Thus SARS-CoV-2 may produce its neurocognitive effects by affecting the neurovascular unit that protects the gut and the brain while simultaneously leading to the increased production of neurotoxic and neuroinflammatory substances in the gut.

Our results are generally aligned with those reported in a nationwide cohort study conducted in Demark during the spring of 2021 (52) which included over 33,000 children. However, despite a high prevalence of symptoms lasting >4 weeks among SARS-CoV-2-positive children (28%), this differed from that among control children by only 0.8%. When comparing the responses related to individual symptoms, SARS-CoV-2-infected children were more likely to report chest pain, dizziness, fatigue, loss of smell and taste, muscle weakness, and respiratory problems. On the other hand, concentration difficulties, cough, diarrhea, fever, headache, muscle and joint pain, and nausea were more commonly reported by uninfected children.

Previous research has indicated that among children with COVID-19 infection, the prevalence of anxiety is higher than in the general population (64). However, as this study lacked a control group, symptoms among infected children could not be compared to those without infection (64). In keeping with our results, which found a greater prevalence of anxiety and/or depression among older relative to younger SARS-CoV-2-negative study participants, the impact of the pandemic on new mental health diagnoses has been reported to be more pronounced in adolescents relative to preadolescents (65). This finding suggests that pandemic restrictions may have been more impactful in the development of mental health disorders than infection itself (66). Potential societal events of the pandemic that may have impacted the mental health of adolescents include school closures, social distancing, economic hardship, and increased time spent on social media (67). Alternatively, it is possible that healthcare providers are less comfortable assigning diagnoses of anxiety and depression to younger children or that such children are less likely to verbalize and/or parents to recognize symptoms of anxiety and depression (68). In addition, clinicians should be cognizant of the association between abdominal pain and functional gastrointestinal disorders and the presence of anxiety and depression, with somatic symptoms reflecting the presence of mental health disorders (69).

Our study has some limitations that should be considered. A significant number of consented participants were lost to follow-up, and those who did complete follow-up were younger, more likely to have been vaccinated, and presented later in the pandemic, thereby potentially introducing bias (Supplementary Table S3). The difference in vaccination rates reflects the fact that many of the excluded children were enrolled early in the study and thus were ineligible for the 6- and/or 12-month follow-up surveys. At the time of their enrollment, near the beginning of the pandemic, vaccines were not available, particularly among younger children.

Our reliance on caregiver-reported data may have led to inaccuracies. Previous research has demonstrated that parents tend to report externalizing problems more precisely than children; however, it is unclear which group reports internalizing symptoms better (70). This may reflect the fact that children value their behavior more positively than parents (71). Although parent–adolescent agreement on emotional and behavioral problems has been reported to be high, adolescents tend to report more problems than their parents (72) and were found to be more sensitive to pain and mental health problems (73). As such our use of parental report may have underestimated the prevalence of anxiety and depression in our teenage population.

We were unable to cross-reference our findings with administrative datasets as most mental healthcare in Canada is provided privately and thus is unavailable in provincially held data repositories. Antibody testing was not performed on participants, and thus we cannot exclude the possibility of infection during or before our follow-up period among control participants. Additionally, as numerous viral illnesses are associated with the development of chronic anxiety and/or depressive symptoms, this may have been the case for some control children (74). As our study was limited to those who speak English or French and we excluded children with pre-existing mental health diagnoses, our findings may not reflect the experiences of the excluded children and their families. In addition, our study population was limited to children seeking ED care, and as such children tend to be sicker than those treated by primary care physicians or who did not seek care at all. This may have biased our SARS-CoV-2 cohort to the inclusion of those with more severe disease (75). The bias toward more severe COVID-19 disease in the acute phase may have further biased our results toward identifying an association between infection and long-term neuropsychiatric symptoms among infected children as hospitalization and severe COVID-19 are associated with an increased risk of developing the post-COVID-19 condition (76).

We could not perform a regression analysis to determine if the association between SARS-CoV-2 infection and new diagnoses of anxiety and/or depression persisted after adjustment for confounders (e.g., sex, chronic conditions, and variants) due to the small number of outcome events. This may be particularly relevant as a greater proportion of SARS-CoV-2-positive participants were male (Table 1), and they were less likely to experience anxiety and depression (77). Lastly, due to pandemic restrictions, there were challenges in accessing healthcare, and people also avoided seeking care. As such, mental healthcare provider access was reduced, and formal diagnoses of anxiety or depression may have been delayed or missed in some participants. Importantly, we did not incorporate symptom severity into our analysis, and we excluded children with pre-existing mental health conditions. Future studies should integrate measures of severity to further our understanding of the effects of SARS-CoV-2 infection on children with pre-existing diagnoses (i.e., whether SARS-CoV-2 infection exacerbated symptoms).

## Conclusion

In this prospective cohort study with 6- and 12-month follow-ups, there was no association between SARS-CoV-2 infection itself and new diagnoses of anxiety and/or depression in children tested for acute infection in an ED setting. This finding, in the context of an increased prevalence of such diagnoses during the pandemic, as noted in our study population of older SARS-CoV-2-negative participants, underscores the complex impacts of broad societal changes on the mental health of children. However, our finding that some non-specific symptoms, such as confusion, abdominal pain, and insomnia were more frequently reported by SARS-CoV-2-positive participants, emphasizes the need for further investigation of the precise pathophysiologic mechanisms underlying the development of chronic symptoms in children.

## Data Availability

Data will only be made available by the authors to requestors with evidence of research ethics board approval and establishment of a data sharing agreement.
