# Supplementary material for: Pediatric SARS-CoV-2 infection and development of anxiety and depression
Source: Front Pediatr. 2025 Mar 17;13:1524617. doi: 10.3389/fped.2025.1524617 (PMC11955925; doi:10.3389/fped.2025.1524617)
Supplement: Supplementary file 1 [file Datasheet1.pdf]

Supplemental Material

eTable 1. Participating study sites and number of participants who completed follow-up.

eTable 2. Modified International Severe Acute Respiratory and Emerging Infection Consortium (ISARIC) Long-COVID Pediatric Questionnaire

eTable 3. Comparison of eligible participants who completed either 6- or 12-month surveys versus those who did not.

eTable 4. Secondary outcomes at 6-month follow-up based on index SARS-CoV-2 test result status.

eTable 5. Secondary outcomes at 12-month follow-up based on index SARS-CoV-2 test result status.

eTable 1. Participating study sites and number of participants who completed follow-up.

Study Site	SARS-CoV-2 Negative	SARS-CoV-2 Positive	Total
Montreal Childrens Hospital	225	48	273
Alberta Childrens Hospital (Calgary)	101	67	168
BC Childrens Hospital (Vancouver)	84	18	102
McMaster Childrens Hospital (Hamilton)	79	14	93
The Childrens Hospital of Winnipeg	65	8	73
IWK Health Centre (Halifax)	56	8	64
CHU Sainte-Justine (Montreal)	21	37	58
Jim Pattison Childrens Hospital (Saskatoon)	40	3	43
The Hospital for Sick Children (Toronto)	12	29	41
Childrens Hospital of Eastern Ontario (Ottawa)	12	25	37
Centre Hospitalier de l'Université de Laval (Québec City)	11	10	21
Janeway Childrens Health and Rehabilitation Centre (St. Johns)	16	0	16
Childrens Hospital London Health Sciences Centre	13	1	14
Kingston Health Sciences Centre	8	0	8

eTable 2. Modified International Severe Acute Respiratory and Emerging Infection Consortium (ISARIC) Long-COVID Pediatric Questionnaire.

Protocol: PERC PHAC COVID-19 Surveillance Network
Site ID: |__|__|__|__| (XXXX) Subject ID: |__|__|__| (XXX)

Patient 6 Month Follow Up Form (CRF 10.0)

Contact Details

Method of first contact attempt *(select only one response)*: ☐ Text ☐ E-mail ☐ Telephone ☐ Other, specify: ___________

Successful: ☐ Yes ☐ No

Method of second contact attempt *(select only one response)*: ☐ Text ☐ E-mail ☐ Telephone ☐ Other, specify: ___________

Successful: ☐ Yes ☐ No

Method of third contact attempt *(select only one response)*: ☐ Text ☐ E-mail ☐ Telephone ☐ Other, specify: ___________

Successful: ☐ Yes ☐ No

Method of fourth contact attempt *(select only one response)*: ☐ Text ☐ E-mail ☐ Telephone ☐ Other, specify: ___________

Successful: ☐ Yes ☐ No

Method of fifth contact attempt *(select only one response)*: ☐ Text ☐ E-mail ☐ Telephone ☐ Other, specify: ___________

Successful: ☐ Yes ☐ No

Return to Medical Care

In the past three months, has your child had any chronic signs/symptoms or diagnoses by a medical professional that may have been related to COVID-19 (if child was positive) or the reason behind their initial ED presentation on date:_____________?

☐ Yes
☐ No chronic signs/symptoms or diagnoses
☐ No, child passed away

If NO chronic signs/symptoms or diagnoses, STOP

If NO, child passed away:

Days after study ED visit that death occurred: |__|__|__| *(days)*

Version Number: 1.0
Version Date: 3/Aug/2021

Was death primarily due to COVID-19 or a complication of COVID-19?

☐ Yes
☐ No
☐ Unknown

Cause of death: ______________________

(Including complications of COVID-19, if applicable)

If YES, chronic respiratory symptoms (e.g., cough, wheezing, shortness of breath):

☐ Yes
☐ No

If YES to chronic respiratory symptoms, please specify: ______________________

If YES, neurological or psychobehavioural symptoms (e.g., anxiety, depression, newfound lack of concentration, poor memory)

☐ Yes
☐ No
☐ Unknown/too young to assess

If YES for neurological or psychobehavioural, please specify: ______________________

If YES, has your child had any other chronic signs/symptoms/diagnoses?

☐ Yes
☐ No

If YES, please specify other chronic signs/symptoms/diagnoses: ______________________

In the last 3 months has your child received COVID-19 vaccine?

☐ Yes
☐ No
☐ Received more than 3 months ago
☐ Unknown

If yes, how many doses?

☐ 1
☐ 2

Dose #1:

- ☐ Pfizer-BioNTech
- ☐ Moderna
- ☐ Other, specify____________

Date dose #1 received:

____________(date format)

☐ Do not recall

If date not recalled: when was dose #1 given?

- ☐ within the last 14 days
- ☐ more than 2 weeks ago
- ☐ > 4 weeks ago
- ☐ Do not recall

Dose #2

- ☐ Pfizer-BioNTech
- ☐ Moderna
- ☐ Other, specify____________

Date dose #2 received:

____________(date format)

☐ Do not recall

If date not recalled: when was dose #2 given?

- ☐ within the last 7 days
- ☐ within the last 2 weeks
- ☐ within the last 2-4 weeks
- ☐ > 4 weeks ago
- ☐ Do not recall

Long-COVID Questionnaire – ISARIC WHO COVID-19

Has your child ever sought support from child /adolescent Mental Health services (psychologist, psychiatrist, social worker, counsellor) before the Covid-19 pandemic?

- ☐ Yes
- ☐ No

Prior to your child coming to the emergency department on ____________ how was your child's physical health in general?

☐ Very poor

☐ Poor

☐ Ok

☐ Good

☐ Very good

If you chose poor or very poor, please explain:

Prior to your child coming to the emergency department on ____________, how would you describe your child's mental /psychological health in general

☐ Very poor

☐ Poor

☐ Ok

☐ Good

☐ Very good

If you chose poor or very poor, please explain:

Has your child visited a doctor/health center because of health consequences related to their emergency department visit on ____________?

☐ Yes

☐ No

Has your child felt feverish recently?

☐ Yes

☐ No

If yes indicate when you felt feverish (tick all that apply)

- ☐ Within the last 7 days
- ☐ 1-2 weeks
- ☐ >2-4 weeks
- ☐ >1-2 months
- ☐ >2-3 months
- ☐ >3-6 months
- ☐ Since illness onset bringing them to the emergency department on ____________

If yes what was the most likely cause of your child's most recent feverish illness?

- ☐ COVID-19
- ☐ Other respiratory infection (cough/cold/sore throat)
- ☐ TB
- ☐ Stomach infection (diarrhea/vomiting)

- ☐ Urinary infection
- ☐ Other (Specify): ________________
- ☐ Unknown
- ☐ Prefer not to say

How much do you agree with the following statement?
"My child has fully recovered from the illness that brought them to the emergency department on ____________"

Please **mark an X** on the line below that match your opinion on the question as of **TODAY:**

0	1	2	3	4	5	6	7	8	9	10
Strongly disagree					Neutral					Strongly agree

How much difficulty is your child having breathing?
Please state the number under
the figure that best describes it:
Number:____________

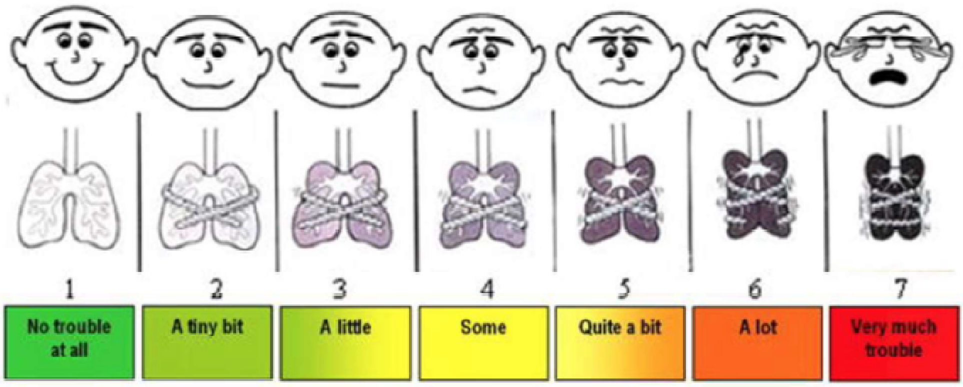

Within the last seven days, has your child had any of these symptoms, which were NOT present before the illness that brought your child coming to the emergency department on ____________? (Indicate if you have a symptom (tick Yes) and also if your child does not have a specific symptom (tick no))

**Nasal congestion/rihnorrhea**	☐ Yes<br>☐ No<br>☐ Unknown
**Difficulty breathing/chest tightness/breathlessness**	☐ Yes<br>☐ No<br>☐ Unknown
**Pain on breathing**	☐ Yes

	☐ No<br>☐ Unknown
**Chest pain**	☐ Yes<br>☐ No<br>☐ Unknown
**Persistent cough**	☐ Yes<br>☐ No<br>☐ Unknown<br>If yes,<br>☐ dry cough<br>☐ with phlegm
**Cannot fully move or control movement**	☐ Yes<br>☐ No<br>☐ Unknown
**Problems with balance**	☐ Yes<br>☐ No<br>☐ Unknown
**Persistent muscle pain**	☐ Yes<br>☐ No<br>☐ Unknown
**Joint pain or joint swelling**	☐ Yes<br>☐ No<br>☐ Unknown
**Headache**	☐ Yes<br>☐ No<br>☐ Unknown
**Dizziness/light headedness**	☐ Yes<br>☐ No<br>☐ Unknown
**Fainting/blackouts**	☐ Yes<br>☐ No<br>☐ Unknown
**Problems seeing/blurred vision**	☐ Yes<br>☐ No<br>☐ Unknown
**Disturbed smell**	☐ Yes<br>☐ No

	☐ Unknown
**Disturbed taste**	☐ Yes<br>☐ No<br>☐ Unknown
**Tremor/shakiness**	☐ Yes<br>☐ No<br>☐ Unknown
**Tingling feeling/"pins and needles"**	☐ Yes<br>☐ No<br>☐ Unknown
**Seizures/fits**	☐ Yes<br>☐ No<br>☐ Unknown
**Confusion/lack of concentration**	☐ Yes<br>☐ No<br>☐ Unknown
**Problems speaking or communicating**	☐ Yes<br>☐ No<br>☐ Unknown
**Insomnia (hard to fall asleep, hard to stay asleep)**	☐ Yes<br>☐ No<br>☐ Unknown
**Hypersomnia (excessive daytime sleepiness or prolonged nighttime sleep)**	☐ Yes<br>☐ No<br>☐ Unknown
**Fatigue**	☐ Yes<br>☐ No<br>☐ Unknown
**Weight loss**	☐ Yes<br>☐ No<br>☐ Unknown
**Problems swallowing or chewing**	☐ Yes<br>☐ No<br>☐ Unknown
**Poor appetite**	☐ Yes<br>☐ No

	☐ Unknown
**Diarrhea**	☐ Yes<br>☐ No<br>☐ Unknown
**Stomach/abdominal pain**	☐ Yes<br>☐ No<br>☐ Unknown
**Feeling nauseous**	☐ Yes<br>☐ No<br>☐ Unknown
**Vomiting**	☐ Yes<br>☐ No<br>☐ Unknown
**Constipation**	☐ Yes<br>☐ No<br>☐ Unknown
**Palpitations (heart racing)**	☐ Yes<br>☐ No<br>☐ Unknown
**Variations in heart rate (Tachycardia or bradycardia)**	☐ Yes<br>☐ No<br>☐ Unknown
**Urination problems**	☐ Yes<br>☐ No<br>☐ Unknown
**Changes in menstruation (if regular before illness that brought your child coming to the emergency department on ___________)**	☐ Yes<br>☐ No<br>☐ Unknown<br>☐ Not applicable
**Bilateral (both eyes) conjunctivitis (red or injected)**	☐ Yes<br>☐ No<br>☐ Unknown<br>If yes,<br>☐ purulent<br>☐ non-purulent
**Skin rash**	☐ Yes<br>☐ No

	☐ Unknown<br>If yes, tick all body areas affected<br>☐ face<br>☐ trunk (stomach or back)<br>☐ arms<br>☐ legs<br>☐ other
**Any other new symptoms, if yes, specify all:**	______________________________

Since the illness that brought your child coming to the emergency department on ____________, has your child been diagnosed with any of the following?

**Multisystem inflammatory syndrome of Children (MIS-C)**	☐ Yes<br>☐ No<br>☐ Unknown
**Pulmonary embolism/micro emboli (PE, Clot in lung)**	☐ Yes<br>☐ No<br>☐ Unknown
**Kawasaki disease**	☐ Yes<br>☐ No<br>☐ Unknown
**Respiratory failure**	☐ Yes<br>☐ No<br>☐ Unknown
**Asthma**	☐ Yes<br>☐ No<br>☐ Unknown
**Reduced lung function**	☐ Yes<br>☐ No<br>☐ Unknown
**Myocarditis (Inflammation of the heart muscle)**	☐ Yes<br>☐ No<br>☐ Unknown
**Depression**	☐ Yes<br>☐ No<br>☐ Unknown
**Anxiety**	☐ Yes<br>☐ No

	☐ Unknown
**Diabetes (if yes indicate type:**<br>☐ Type 1<br>☐ Type 2	☐ Yes<br>☐ No<br>☐ Unknown
**Shock/toxic shock syndrome**	☐ Yes<br>☐ No<br>☐ Unknown
**Coagulopathy (excessive bleeding or clotting)**	☐ Yes<br>☐ No<br>☐ Unknown
**Kidney problems**	☐ Yes<br>☐ No<br>☐ Unknown
**Intussusception**	☐ Yes<br>☐ No<br>☐ Unknown
**Other (please indicate)**	________________________________

Your child's overall health status

We would like to know how good or bad your child's health is

- Line is numbered 0 to 100
- 100% means the best health you can imagine
- 0% means the worst health you can imagine
- Please mark an X on the line that shows how good or bad your child's health is TODAY and how it was BEFORE their COVID-19 illness

eTable 3. Comparison of eligible participants who completed either 6- or 12-month surveys versus those who did not.

Participants characteristics	Did not complete either 6- or 12-month follow-up (n=1326)	Completed either 6- or 12-month follow-up (n=1011)	P
Age, years, median (IQR)	8.0 (5.0, 13.0)	7.0 (5.0, 11.0)	<0.001
Age group, n (%)			<0.001
4 – <12	923/1326 (69.6%)	817/1011 (80.8%)	
12 – <18	403/1326 (30.4%)	194/1011 (19.2%)	
Asthma, n (%)	265/1324 (20.0%)	188/1009 (18.6%)	0.40
COVID Vaccination received prior to index ED visit, n (%)§	33/366 (9.0%)	120/662 (18.1%)	<0.001
Hospitalized for the acute illness, n (%)†	179/1326 (13.5%)	123/1011 (12.2%)	0.34
Indigenous, n (%)	92/1315 (7.0%)	64/1004 (6.4%)	0.55
Number of symptoms at baseline, median (IQR)	5.0 (3.0, 8.0)	6.0 (3.0, 8.0)	0.03
Preexisting chronic condition,* n (%)	268/1324 (20.2%)	190/1009 (18.8%)	0.40
Severe acute illness outcome, n (%)†‡	15/1324 (1.1%)	10/1006 (1.0%)	0.75
Sex, male, n (%)	699/1326 (52.7%)	548/1011 (54.2%)	0.48
Variant, n (%)**			<0.001
Wild type	987 (74.4%)	266 (26.3%)	
Alpha	179 (13.5%)	198 (19.6%)	
Beta	1 (0.1%)	0 (0%)	
Gamma	2 (0.2%)	0 (0%)	
Delta	118 (8.9%)	384 (38.0%)	
Omicron	39 (2.9%)	163 (16.1%)	

* Excluding asthma.
§ The question was implemented to the study on June 11, 2021, about 10 months after the study was officially launched.
** For SARS-CoV-2 negative group coded as: Wild type (before April 18, 2021), Alpha (April 18-June 26, 2021), Delta (June 27, 2021-Dec 11, 2021), Omicron (Dec 12, 2021-Present day)
† At or within 14 days of the index ED visit.
‡ A severe outcome reflected the occurrence of a life-threatening complication or performance of an intervention required to address a potentially life-threatening event. [1]

eTable 4. Secondary outcomes at 6-month follow-up based on index SARS-CoV-2 test result status.

Symptomatology	All N=433	SARS-CoV-2 Negative N=278	SARS-CoV-2 Positive N=155	Difference of % (95%CI) *	Unadjusted Odds ratio (95%CI)	Adjusted p value*
Anxiety and/or Depression	9/433 (2.1)	6/278 (2.2)	3/155 (1.9)	-0.2 (-3.02, 3.1)	0.9 (0.2, 3.6)	>0.99
Depression	3/433 (0.7)	2/278 (0.7)	1/155 (0.6)	-0.1 (-1.93, 2.3)	0.9 (0.1, 10.0)	>0.99
Anxiety	8/433 (1.8)	6/278 (2.2)	2/155 (1.3)	-0.9 (-3.41, 2.23)	0.6 (0.1, 3.0)	0.99
Chest pain	6/434 (1.4)	3/278 (1.1)	3/156 (1.9)	0.8 (-1.7, 3.9)	1.8 (0.4, 9.0)	0.99
Confusion/lack of concentration	3/434 (0.7)	0/278 (0)	3/156 (1.9)	1.9 (-0.4, 4.7)	n/a	0.35
Dizziness	7/434 (1.6)	2/278 (0.7)	5/156 (3.2)	2.5 (-0.5, 5.9)	4.6 (0.9, 23.8)	0.35
Fainting	2/434 (0.5)	1/278 (0.4)	1/156 (0.6)	0.3 (-1.5, 2.6)	1.8 (0.1, 28.8)	>0.99
Fatigue	17/434 (3.9)	6/278 (2.2)	11/156 (7.1)	4.9 (0.6, 9.6)	3.4 (1.25, 9.49)	0.35
Headache	27/434 (6.2)	16/278 (5.8)	11/156 (7.1)	1.3 (-3.5, 6.5)	1.2 (0.6, 2.8)	0.99
Hypersomnia	3/434 (0.7)	2/278 (0.7)	1/156 (0.6)	-0.1 (-1.9, 2.3)	0.9 (0.1, 10.0)	>0.99
Insomnia	11/434 (2.5)	5/278 (1.8)	6/156 (3.8)	2.1 (-1.3, 5.9)	2.2 (0.7, 7.3)	0.5
Joint pain	7/434 (1.6)	4/278 (1.4)	3/156 (1.9)	0.5 (-2.2, 3.7)	1.3 (0.3, 6.1)	0.99
Muscle pain	10/434 (2.3)	6/278 (2.2)	4/156 (2.6)	0.4 (-2.6, 4.0)	1.2 (0.3, 4.3)	0.99
Nauseous	12/434 (2.8)	6/278 (2.2)	6/156 (3.8)	1.7 (-1.8, 5.6)	1.8 (0.6, 5.7)	0.76
Palpitations	3/434 (0.7)	2/278 (0.7)	1/156 (0.6)	-0.1 (-1.9, 2.3)	0.9 (0.1, 9.9)	>0.99
Poor appetite	20/434 (4.6)	12/278 (4.3)	8/156 (5.1)	0.8 (-3.3, 5.4)	1.2 (0.5, 3.0)	0.99

Problems seeing	4/434 (0.9)	1/278 (0.4)	3/156 (1.9)	1.56 (-0.8, 4.5)	5.4 (0.6, 52.7)	0.35
Problems with balance	2/434 (0.5)	0/278 (0)	2/156 (1.3)	1.3 (-0.7, 3.8)	n/a	0.35
Stomach/abdominal pain	22/434 (5.1)	10/278 (3.6)	12/156 (7.7)	4.1 (-0.6, 9.2)	2.2 (0.9, 5.3)	0.35
Tingling	0/434 (0)	0/278 (0)	0/156 (0)	0 (-1.1, 1.7)	n/a	n/a
Tremor	2/434 (0.5)	0/278 (0)	2/156 (1.3)	1.3 (-0.7, 3.8)	n/a	0.35
Weight loss	4/434 (0.9)	1/278 (0.4)	3/156 (1.9)	1.6 (-0.8, 4.5)	5.3 (0.6, 52.7)	0.35

***** P value**s** were obtained from Chi-square or Fisher's exact tests as appropriate; P values were adjusted via Benjamini- Hochberg approach for multiple comparisons.

eTable 5. Secondary outcomes at 12-month follow-up based on index SARS-CoV-2 test result status.

Symptomatology	All<br><br>N=901	SARS-CoV-2-Negative<br><br>N=674	SARS-CoV-2-Positive<br><br>N=227	Difference of % (95%CI) *	Unadjusted Odds ratio (95%CI)	Adjusted p value
Anxiety and/or Depression	24/901 (2.7)	16/674 (2.4)	8/227 (3.5)	1.15 (-1.36, 4.20)	1.50 (0.63, 3.6)	0.59
Depression	7/901 (0.8)	4/674 (0.6)	3/227 (1.3)	0.73 (-0.81, 2.82)	2.24 (0.5, 10.1)	0.59
Anxiety	23/901 (2.6)	16/674 (2.4)	7/227 (3.1)	0.71 (-1.68, 3.63)	1.31 (0.53, 3.22)	0.81
Chest pain	14/901 (1.6)	9/674 (1.3)	5/227 (2.2)	0.87 (-1.12, 3.40)	1.7 (0.6, 5.0)	0.59
Confusion/lack of concentration	18/901 (2.0)	9/674 (1.3)	9/227 (4.0)	2.63 (0.09, 5.69)	3.1 (1.2, 7.8)	0.18
Dizziness	17/901 (1.9)	12/674 (1.8)	5/227 (2.2)	0.42 (-1.62, 3.01)	1.2 (0.4, 3.6)	0.90
Fainting	6/901 (0.7)	5/674 (0.7)	1/227 (0.4)	-0.30 (-1.41, 1.38)	0.6 (0.1, 5.1)	>0.99
Fatigue	51/901 (5.7)	39/674 (5.8)	12/227 (5.3)	-0.50 (-3.90, 2.90)	0.9 (0.5, 1.8)	0.90
Headache	63/901 (7)	43/674 (6.4)	20/227 (8.8)	2.43 (-1.69, 6.55)	1.42 (0.82, 2.47)	0.59
Hypersomnia	14/901 (1.6)	9/674 (1.3)	5/227 (2.2)	0.87 (-1.12, 3.40)	1.7 (0.6, 5.0)	0.59
Insomnia	30/900 (3.3)	16/674 (2.4)	14/226 (6.2)	3.82 (0.64, 7.49)	2.7 (1.3, 5.7)	0.06
Joint pain	20/901 (2.2)	14/674 (2.1)	6/227 (2.6)	0.57 (-1.65, 3.33)	1.3 (0.5, 3.4)	0.81
Muscle pain	27/901 (3)	21/674 (3.1)	6/227 (2.6)	-0.47 (-2.8, 2.4)	0.8 (0.3, 2.1)	0.91
Nauseous	26/900 (2.9)	15/674 (2.2)	11/226 (4.9)	2.64 (-0.22, 6.01)	2.25 (1.0, 5.0)	0.28
Palpitations	9/901 (1)	7/674 (1)	2/227 (0.9)	-0.16 (-1.56, 1.81)	0.9 (0.2, 4.1)	>0.99
Poor appetite	43/901 (4.8)	29/674 (4.3)	14/227 (6.2)	1.86 (-1.45, 5.67)	1.5 (0.8, 2.8)	0.59
Problems seeing	5/901 (0.6)	3/674 (0.4)	2/227 (0.9)	0.44 (-0.86, 2.30)	2.0 (0.3, 12.0)	0.81
Problems with balance	5/901 (0.6)	5/674 (0.7)	0/227 (0)	-0.74 (-1.56, 0.66)	n/a	0.59

Stomach/abdominal pain	52/901 (5.8)	28/674 (4.2)	24/227 (10.6)	6.42 (2.14, 10.69)	2.7 (1.6, 4.8)	0.007
Tingling	2/901 (0.2)	0/674 (0)	2/227 (0.9)	0.88 (-0.34, 2.66)	n/a	0.28
Tremor	5/901 (0.6)	5/674 (0.7)	0/227 (0)	-0.74 (-1.56, 0.66)	n/a	0.59
Weight loss	22/901 (2.4)	14/674 (2.1)	8/227 (3.5)	1.45 (-1.04, 4.46)	1.7 (0.7, 4.2)	0.59

***** P value**s** were obtained from Chi-square or Fisher's exact tests as appropriate; P values were adjusted via Benjamini- Hochberg approach for multiple comparisons.

References

1. Funk AL, Florin TA, Kuppermann N, et al. Outcomes of SARS-CoV-2-Positive Youths Tested in Emergency Departments: The Global PERN-COVID-19 Study. *JAMA Netw Open*. Jan 4 2022;5(1):e2142322. doi:10.1001/jamanetworkopen.2021.42322
